# Ovarectomy despite Negative Imaging in Anti-NMDA Receptor Encephalitis: Effective Even Late

**DOI:** 10.1155/2013/843192

**Published:** 2013-02-26

**Authors:** Anna-Lena Boeck, Frank Logemann, Terence Krauß, Kais Hussein, Eva Bültmann, Corinna Trebst, Martin Stangel

**Affiliations:** ^1^Department of Neurology, Hannover Medical School, Carl-Neuberg-Straße 1, 30625 Hannover, Germany; ^2^Department of Anaesthesiology and Intensive Care Medicine, Hannover Medical School, Carl-Neuberg-Straße 1, 30625 Hannover, Germany; ^3^Institute of Pathology, Hannover Medical School, Carl-Neuberg-Straße 1, 30625 Hannover, Germany; ^4^Department of Neuroradiology, Hannover Medical School, Carl-Neuberg-Straße 1, 30625 Hannover, Germany

## Abstract

Anti-NMDA receptor (NMDAR) encephalitis is an autoimmune antibody-mediated neuropsychiatric disorder. The disorder is known to be associated with ovarian teratoma and predominantly affects young women. Here, we report the case of a 34-year-old woman with anti-NMDAR encephalitis, in which detailed investigations gave no specific hint for an ovarian teratoma. Despite this, and due to a continuous severe clinical syndrome, an ovarectomy was performed and histological examination revealed an occult teratoma. The ovarectomy led to a remarkable improvement even with a long term intensive care treatment for 11 months. The most important lesson to be learned from this instructive case is that even though none of the investigations was indicative for an ovarian teratoma, including an explorative laparoscopy with biopsy, there still may be an occult ovarian teratoma. This shows that tumour search and diagnosis are extremely important in patients presenting with anti-NMDAR encephalitis, and a laparotomy and ovarectomy is justified. Furthermore, removal of the teratoma even 11 months after a very severe course is still therapeutically effective.

## 1. Introduction

Anti-NMDA receptor (NMDAR) encephalitis represents an autoimmune antibody-mediated form of limbic encephalitis first described in 2007 [1]. The typical clinical picture presents with neuropsychiatric symptoms, seizures, dyskinesias, and autonomic dysregulation. Many case reports and case series have widened the clinical spectrum from mild manifestations with only few symptoms and fast recovery to dramatic cases with the requirement of many months of intensive care treatment [1–3]. Predominantly young women are affected in many of whom an underlying tumour, mostly an ovarian teratoma, is detected [1–3]. These cases are thought to be paraneoplastic, in particular due to the rapid clinical improvement after ovarectomy. However, in some cases, no teratoma or other tumour can be found. In other cases the presentation preceded the tumour manifestation by years [4].

## 2. Case Presentation

**Figure 1:**
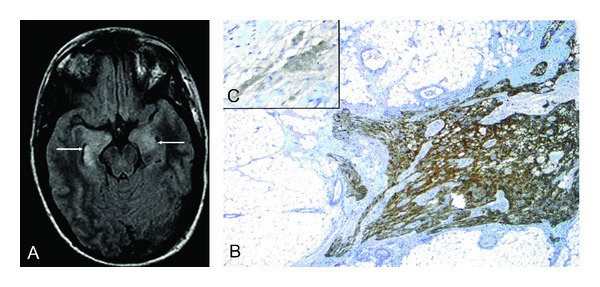
Axial FLAIR magnetic resonance (MR) imaging (A) shows bilateral hyperintense signal in the mildly swollen medial temporal lobe (arrows). Histological examination (B and C) of the resected ovar revealed a cystic teratoma of 2.9 cm diameter including focal neuronal differentiation of ~0.5 cm diameter. Immunohistochemistry of glial fibrillary acidic protein (B; magnification ×25) and *neuron* specific enolase (C; magnification ×400) underlined the presence of focal glial and partial neuronal differentiation. Other parts of the teratoma showed squamous epithelial differentiation with hair follicles, sweat as well as sebaceous glands and fat/connective tissue, respiratory epithelia, cartilage as well as osteocytes with bone matrix, and also haematopoiesis.

We treated a 34-year-old woman with no remarkable medical history who presented with life-threatening hyperkinesias, autonomic dysfunction, hypoventilation, and epileptic status. Further diagnostic work-up showed temporomesial abnormalities on magnetic resonance imaging (MRI) of the brain (Figure 1). Cerebrospinal fluid (CSF) analysis revealed inflammatory changes with a pleocytosis of 154 leukocytes/*μ*l, predominantly activated lymphocytes with 35% plasma cells, and elevated albumin quotient of 10.2 indicating a mild blood-brain-barrier impairment and detection of oligoclonal bands. Based on typical clinical presentation [1] and supportive MRI and CSF findings the diagnosis of limbic encephalitis was made. Immunotherapies were immediately started, even before laboratory confirmation of the presence of anti-N-methyl D-aspartate (NMDA) receptor antibodies was available.

Treatments for our patient included sequentially high dose steroids (5 × 1 g methylprednisolone IV), intravenous immunoglobulin (IVIg, 5 × 0.4 g/kg), plasma exchange (5 cycles), rituximab (4 × 375 mg), and cyclophosphamide (750 mg/m^2^ body surface area). At first presentation, a thorough search for a tumour was performed including repeated high resolution computed tomography (CT) of the abdomen, pelvis, and chest; positron emission tomography with fluorodeoxyglucose imaging (FDG-PET) of the whole body; and gynaecological ovarian ultrasound and explorative laparoscopy including biopsy of both ovaries. The only finding was a minimal suspect lesion in the right ovary on ultrasound examination. None of the immunological therapies had any effect on the severity of symptoms and intensive care treatment was required for several months. Due to the therapeutic resistance and despite the negative search for an ovarian teratoma, we nevertheless decided to perform an ovarectomy of the right side 11 months after initial presentation. This revealed a mature teratoma with partial neuronal differentiation (Figure 1). Clinical improvement started days after removal of the teratoma and continued slowly. 12 months after ovarectomy the patient shows reduced cognitive functions as well as a generally reduced psychomotoric status but without dyskinesias, seizures, or spasms. She is able to communicate in short sentences.

## 3. Discussion

In several aspects our case of anti-NMDAR encephalitis is very typical. An otherwise healthy young woman develops rapidly severe hyperkinesias and an epileptic status, which immediately led to the search for anti-NMDAR antibodies. Therefore, the correct diagnosis in our case was easy to make. The further treatment and disease course, however, was rather difficult and long lasting. The patient was treated with several different immunosuppressive agents including the monoclonal antibody rituximab and cyclophosphamide. Despite such intensive therapeutic regime the clinical course was continuously progressing and required intensive care treatment over more than a year. The intensive search for an underlying teratoma revealed no such even, though a diagnostic laparoscopy and bilateral ovarian biopsy was performed. The decision to perform an ovarectomy with no diagnostic hints for a teratoma was made in the view of a deteriorating clinical condition and in a multidisciplinary approach. The final finding of an occult teratoma and the rapid clinical improvement was rewarding for the treating team and the patient's family. This experience has been made by others [5, 6]. Johnson et al. describe a case of a 35-year-old women with anti-NMDAR encephalitis in nonconvulsive status epilepticus lasting 6 months with marked improvement following the removal of an ovarian teratoma. Clearly, the prognosis is better if the tumour is identified and removed early in the disease course [2] but these cases show that even a late removal can still lead to a clinical improvement. Other case series have reported that severe anti-NMDAR encephalitis can be potentially reversible even without removing the tumour and immunotherapy can be partially effective [4]. However overall, if the tumour is not removed the chance of recovery is lower, the course of disease longer, and the frequency of relapses higher.

## 4. Conclusion

In conclusion, there are several important lessons to be learned from this instructive case with anti-NMDAR encephalitis.

1. Even though none of the investigations was indicative for an ovarial teratoma, including an explorative laparoscopy with biopsy, there still may be an occult ovarian teratoma. This shows that tumour search and diagnosis are extremely important in patients presenting with anti-NMDAR encephalitis and a laparotomy and ovarectomy is justified [4].
2. Removal of the teratoma even 11 months after a very severe course is still therapeutically effective. Thus, intensive care treatment should be maintained even for many months since the outcome may still be favourable as also shown in a case described by Johnson et al. [5].
